# Conveniently-Grasped Field Assessment Stroke Triage (CG-FAST): A Modified Scale to Detect Large Vessel Occlusion Stroke

**DOI:** 10.3389/fneur.2019.00390

**Published:** 2019-04-17

**Authors:** Xiaoxian Gong, Zhicai Chen, Feina Shi, Meixia Zhang, Chao Xu, Ruiting Zhang, Min Lou

**Affiliations:** ^1^Department of Neurology, The Second Affiliated Hospital of Zhejiang University, School of Medicine, Hangzhou, China; ^2^Department of Neurology, Jinhua Hospital of Zhejiang University, Jinhua, China; ^3^Zhejiang University Brain Research Institute, Hangzhou, China

**Keywords:** large artery occlusion, stroke, endovascular treatment, NIHSS, scale

## Abstract

**Background and Purpose:** Patients with large vessel occlusion stroke (LVOS) need to be rapidly identified and transferred to comprehensive stroke centers (CSC). However, previous pre-hospital strategy remains challenging. We aimed to develop a modified scale to better predict LVOS.

**Methods:** We retrospectively reviewed our prospectively collected database for acute ischemic stroke (AIS) patients who underwent CT angiography (CTA) or time of flight MR angiography (TOF-MRA) and had a detailed National Institutes of Health Stroke Scale (NIHSS) score at admission. Large vessel occlusion (LVO) was defined as the complete occlusion of large vessels, including the intracranial internal carotid artery (ICA), M1, and M2 segments of the middle cerebral artery (MCA), and basilar artery (BA). The Conveniently-Grasped Field Assessment Stroke Triage (CG-FAST) scale consisted of Level of Consciousness (LOC) questions, Gaze deviation, Facial palsy, Arm weakness, and Speech changes. Receiver Operating Characteristic (ROC) analysis was used to obtain the Area Under the Curve (AUC) of CG-FAST and previously established pre-hospital prediction scales.

**Results:** Finally, 1,355 patients were included in the analysis. LVOS was detected in 664 (49.0%) patients. The sensitivity, specificity, positive predictive value, and negative predictive value of CG-FAST were 0.617, 0.810, 0.785, and 0.692 respectively, at the optimal cutoff (≥4). The AUC, Youden index and accuracy of the CG-FAST scale (0.758, 0.428, and 0.728) were all higher than other pre-hospital prediction scales.

**Conclusions:** CG-FAST scale could be an effective and simple scale for accurate identification of LVOS among AIS patients.

## Introduction

Large vessel occlusion stroke (LVOS) often leads to severe disability and mortality. Although endovascular therapy (EVT) has been proved to be effective for LVOS patients (1–9), especially in anterior circulation, its benefit is highly time-dependent (10, 11). As hospitals with around-the-clock endovascular capability are scarce in many parts of the world and patients admitted directly to a CSC would have better outcomes than those receiving drip and ship treatment (12, 13), this requirement presents a big challenge to current systems of delivering appropriate patients to comprehensive stroke centers (CSC). It is thus critical for emergency paramedics to precisely identify LVOS in the setting of pre-hospital triage stage (14, 15).

Current guideline recommends the use of National Institutes of Health Stroke Scale (NIHSS) scores ≥6 to select patients for EVT (16), as patients with acute LVOS are strongly associated with high NIHSS (17–19). However, in the pre-hospital triage stage, it is not very practical to complete the whole examination of NIHSS. Actually, certain items of NIHSS may be more informative of LVOS than a simple score, which usually reflects the overall severity of deficits. Thus, several examination tools derived from NIHSS have been proposed for use in the pre-hospital setting (14, 20–25), for example, recent G-FAST score (Gaze, Face, Arm, and Speech) has been shown to improve the predictive capability of LVOS when compared to other tools (25). Based on our clinical observation, we found that the level of consciousness, which was not included in G-FAST, was easier to be disturbed in LVOS patients and it had relatively high specificity to indicate the presence of LVOS when combined with other neurological deficits. We posit the addition of the item “consciousness level” to the G-FAST score may increase the predictive ability. Thus, we developed a new simple scale derived from the G-FAST score, named the Conveniently-Grasped Field Assessment Stroke Triage (CG-FAST) scale and then designed a test to determine whether the CG-FAST scale could achieve a better predictive performance than the preexisting scales for accurate identification of LVOS.

## Materials and Methods

### Subjects and Methods

We retrospectively reviewed our prospectively collected database for acute stroke patients (hemorrhagic or ischemic stroke) within 8 h of onset in our center during period from June 2009 to July 2018. We enrolled patients who (1) underwent CT angiography (CTA) or time of flight MR angiography (TOF-MRA) within 8 h, (2) had a detailed NIHSS at admission, (3) had a diagnosis of AIS confirmed by diffusion-weighted imaging or CT at 24 h after symptom onset. Patients with poor image quality due to motion artifacts were excluded. Demographic, clinical, and laboratory data were recorded including age, gender; prior antiplatelet therapy; risk factors (smoking, hypertension, diabetes mellitus, hyperlipidemia, history of stroke/TIA, and atrial fibrillation); blood pressure and blood glucose.

As shown in Table 1, the CG-FAST scale (*L*OC Questions [scored 0–1], *G*aze [0–1], *F*acial palsy [scored 0–1], *A*rm weakness [0–1], and *S*peech problems [0–1]) was graded as detailed and derived from the NIHSS assessed by experienced neurologists at hospital admission.

**Table 1:** The CG-FAST scale and its correspondence to the NIHSS.

**Item**	**CG-FAST score**	**NIHSS score source**
**LOC QUESTIONS**		
Normal	0	0
One correct or neither correct	1	1–2
**GAZE**		
Normal	0	0
Partial or forced deviation	1	1–2
**FACIAL PALSY**		
Normal	0	0
Minor, partial, or complete paralysis	1	1–3
**ARM WEAKNESS**		
No drift or drift but doesn't hit bed	0	0–1
Some effort against gravity, no effort against gravity or no movement	1	2–4
**SPEECH PROBLEMS**		
Normal	0	0
Aphasia or dysarthria	1	1–3/1–2

*CG-FAST indicates Conveniently-Grasped Field Assessment Stroke Triage; LOC, level of consciousness; and NIHSS, National Institutes of Health Stroke Scale*.

LVOS in current study was defined as unilateral occlusion of intracranial internal carotid artery (ICA), M1, and M2 segments of the middle cerebral artery (MCA), and basilar artery (BA) (14, 26) on baseline CTA or TOF-MRA. Two experienced neurologists blinded to the patients' information assessed the occlusion on CTA or TOF-MRA at admission with rater discrepancies settled by consensus.

### Statistical Methods

Patients were dichotomized into a LVOS group and a non-LVOS group. Clinical characteristics were summarized by computing the mean ± standard deviation (SD) or median (interquartile range), and differences between the two groups were estimated by the *t*-test or Mann-Whitney *U*-test if they were continuous variables. Categorical or binary datum was summarized by proportion (n); and differences between the two groups were estimated by the Pearson χ^2^ test. Receiver operating characteristic (ROC) analysis was used to get the area under the curve (AUC) of each pre-hospital prediction scale. The optimal cutoff was determined at the maximal Youden Index (27). Sensitivity, specificity, positive predictive value (PPV), negative predictive value (NPV), and accuracy were calculated for the prediction of LVOS. All statistical analysis was performed using SPSS, Version 22.0 (IBM, Armonk, New York). A *P* < 0.05 was considered statistically significant.

## Results

**Figure 1:**
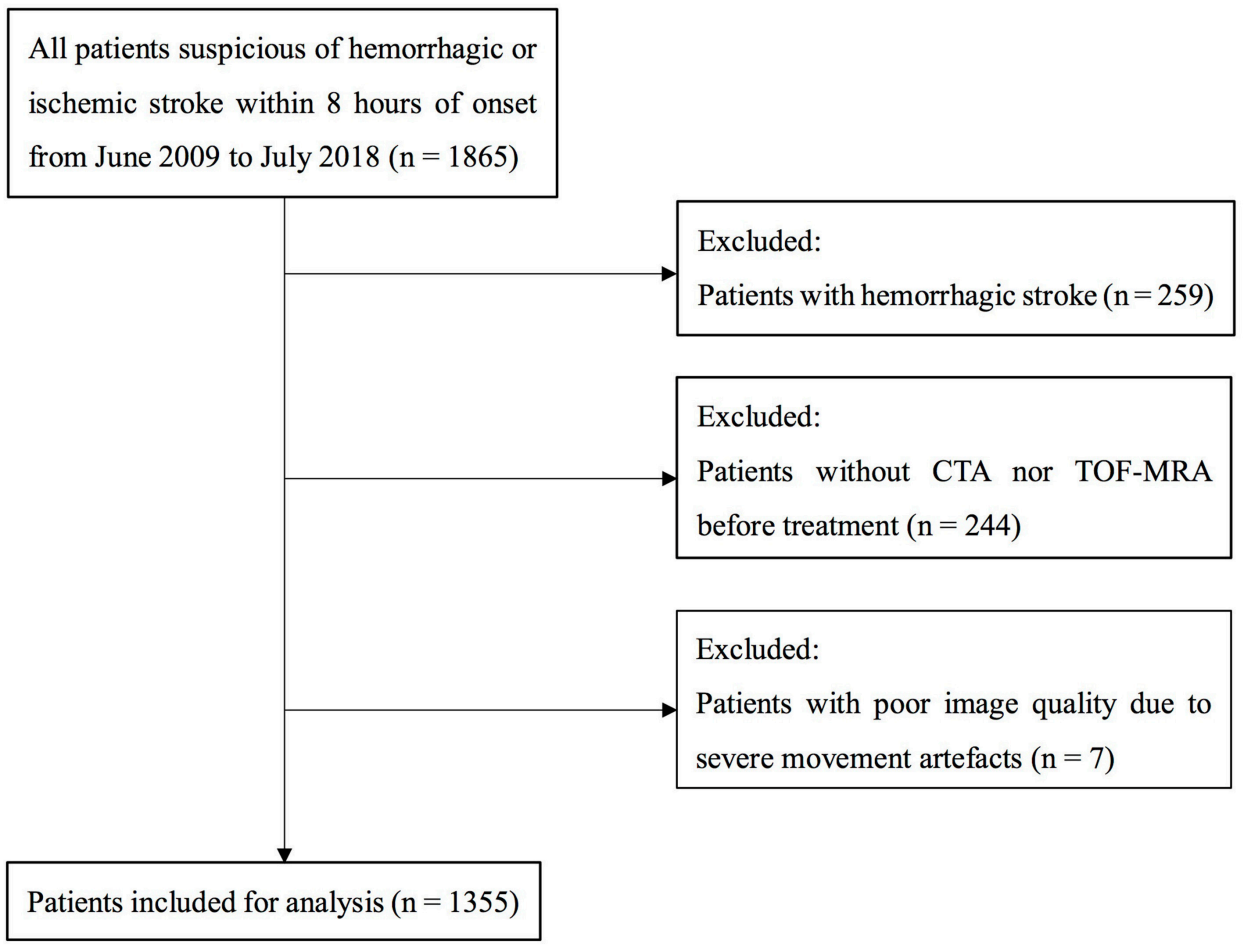
Flow chart of patient selection. CTA indicates CT angiography; and TOF-MRA, time of flight MR angiography.

A total of 1355 patients were finally included in the analysis. The flow chart of patient selection is illustrated in Figure 1. Of the included patients, mean age was 69 ± 13 years and 821 (60.6%) were male. Median NIHSS on admission was 8 (3–15). Among them, 664 (49.0%) patients had LVOS and 691 (51.0%) were in the non-LVOS group.

Table 2 shows that patients with LVOS were older (*p* = 0.001), had higher rate of atrial fibrillation (*p* < 0.001), lower rate of diabetes mellitus (*p* = 0.018), lower systolic blood pressure (*p* < 0.001), lower blood glucose (*p* = 0.025), and higher NIHSS score (*p* < 0.001), compared with non-LVOS patients. Besides, the scores of LOC questions, gaze, facial palsy, arm weakness, and speech changes were significantly higher in the LVOS group.

**Table 2:** Comparison of baseline variables between LVOS and non-LVOS patients.

	**LVOS (*n* = 664)**	**Non-LVOS (*n* = 691)**	***P*-value**
Male (*n*, %)	380 (57.2%)	441 (63.8%)	0.013
Age (year)	70 ± 12	68 ± 13	0.001
Prior antiplatelet therapy (*n*, %)	124 (18.7%)	121 (17.5%)	0.578
Smoking (*n*, %)	194 (29.2%)	234 (33.9%)	0.066
Hypertension (*n*, %)	456 (68.7%)	464 (67.1%)	0.548
Diabetes mellitus (*n*, %)	129 (19.4%)	171 (24.7%)	0.018
Hyperlipidemia (*n*, %)	241 (36.3%)	284 (41.1%)	0.070
History of stroke/TIA (*n*, %)	119 (17.9%)	135 (19.5%)	0.446
Atrial fibrillation (*n*, %)	317 (47.7%)	157 (22.7%)	<0.001
Systolic blood pressure (mmHg)	153 ± 27	159 ± 25	<0.001
Glucose (mmol/L)	7.6 ± 2.6	7.9 ± 2.9	0.025
NIHSS sum	13 (8–18)	4 (2–10)	<0.001
**LOC**			
A) LOC responsiveness	0 (0–1)	0 (0–0)	<0.001
B) LOC questions	1 (0–2)	0 (0–0)	<0.001
C) LOC commands	0 (0–2)	0 (0–0)	<0.001
Gaze deviation	1 (0–2)	0 (0–0)	<0.001
Visual field test	0 (0–0)	0 (0–0)	0.800
Facial palsy	1 (1–2)	1 (0–1)	<0.001
Motor arm	4 (2–4)	0 (0–3)	<0.001
Motor left arm	0 (0–4)	0 (0–1)	<0.001
Motor right arm	0 (0–4)	0 (0–1)	<0.001
Motor leg	3 (2–4)	1 (0–3)	<0.001
Motor left leg	0 (0–3)	0 (0–1)	<0.001
Motor right leg	0 (0–3)	0 (0–1)	<0.001
Limb ataxia	0 (0–0)	0 (0–0)	0.002
Sensory	0 (0–1)	0 (0–1)	0.123
Aphasia	2 (0–3)	0 (0–1)	<0.001
Dysarthria	1 (0–2)	1 (0–1)	<0.001
Extinction and inattention	0 (0–0)	0 (0–0)	0.003
**INFARCTION LOCALIZATION**			
Anterior circulation	594 (89.5%)	591 (85.5%)	0.029
Posterior circulation	61 (9.2%)	81 (11.7%)	0.128
Both involved	9 (1.4%)	19 (2.7%)	0.071
**OCCLUSION SITES**			
ICA	196 (29.5%)	–	–
M1	452 (68.1%)	–	–
M2	32 (4.8%)	–	–
BA	62 (9.3%)	–	–

*LOC, indicates level of consciousness; LVOS, large vessel occlusion strokes; NIHSS, national institutes of health stroke scale; ICA, intracranial internal carotid artery; M1, M1 segment of the middle cerebral artery; M2, M2 segment of the middle cerebral artery; and BA, basilar artery*.

**Figure 2:**
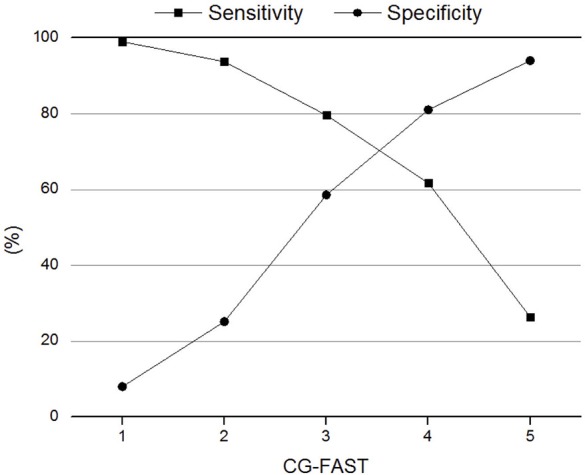
Sensitivity and specificity of different cutoff values of the Conveniently-Grasped Field Assessment Stroke Triage (CG-FAST) scale for the detection of large vessel occlusion stroke.

The AUC of CG-FAST scale was 0.758. Figure 2 shows different cutoff values of the CG-FAST for predicting LVOS. Highest Youden Index was achieved for score ≥ 4, with sensitivity, specificity, PPV and NPV of 0.617, 0.810, 0.785, and 0.692, respectively. The diagnostic parameters of CG-FAST were compared with preexisting scales including FAST-ED, 3I-SS, CPSSS, PASS, RACE, LAMS, G-FAST, and NIHSS (Table 3). The AUC and highest Youden index of the CG-FAST scale were higher than other preexisting scales.

**Table 3:** Comparison of thresholds of the pre-hospital stroke scales, CG-FAST, and NIHSS according to AUC, sensitivity, specificity, PPV and NPV, and accuracy.

**Scale**	**AUC**	**Youden index**	**SEN**	**SPE**	**PPV**	**NPV**	**Accuracy**	**95% CI**	***P*-value**
FAST-ED≥3	0.747	0.401	0.735	0.666	0.714	0.737	0.725	0.719–0.776	<0.001
3-ISS≥3	0.729	0.388	0.587	0.801	0.771	0.680	0.714	0.701–0.758	<0.001
CPSSS≥2	0.751	0.414	0.693	0.721	0.740	0.722	0.730	0.723–0.779	<0.001
PASS≥2	0.739	0.417	0.696	0.721	0.739	0.723	0.731	0.711–0.768	<0.001
RACE≥5	0.740	0.407	0.675	0.732	0.737	0.709	0.722	0.711–0.768	<0.001
LAMS≥3	0.709	0.345	0.785	0.560	0.663	0.696	0.528	0.680–0.739	<0.001
G-FAST≥3	0.715	0.315	0.751	0.564	0.660	0.721	0.687	0.685–0.744	<0.001
CG-FAST≥4	0.758	0.428	0.617	0.810	0.785	0.692	0.728	0.731–0.786	<0.001
NIHSS≥9	0.746	0.416	0.732	0.684	0.719	0.264	0.490	0.718–0.775	<0.001
NIHSS≥6^*^	0.746	0.307	0.818	0.489	0.641	0.761	0.686	0.718–0.775	<0.001

*3I-SS, 3-item Stroke Scale; AUC, area under the curve; CG-FAST, Conveniently-Grasped Field Assessment Stroke Triage; CPSSS, Cincinnati Pre-hospital Stroke Severity scale; CI, confidence interval; FAST-ED, Field Assessment Stroke Triage for Emergency Destination scale; G-FAST, gaze–face–arm–speech–time test; LAMS, Los Angeles Motor Scale; NIHSS, National Institutes of Health Stroke Scale; NPV, negative predictive value; PASS, Pre-hospital Acute Stroke Severity scale; PPV, positive predictive value; and RACE, Rapid Arterial Occlusion Evaluation Scale; SEN, Sensitivity; SPE, Specificity*.

* *NIHSS≥6 is an inclusion criterion for endovascular treatment according to 2018 guidelines for the early management of patients with acute ischemic stroke: a guideline for healthcare professionals from the American Heart Association/American Stroke Association (16)*.

## Discussion

The current study indicated that our novel CG-FAST scale could be effective for accurate identification of LVOS among AIS patients, as its predictive value for LVOS was even higher than other preexisting scales, as well as NIHSS.

There are several advantages of the CG-FAST scale over preexisting scales, which may explain its relatively high predictive value for LVOS. The evaluation of LOC is relatively complicated for emergency paramedics, especially when patients present with somnolence, sopor state or coma. We thus adopted LOC questions in the CG-FAST, which is easier and could be combined with the evaluation of aphasia and dysarthria. Actually, most of the previous pre-hospital scales ignored the evaluation of aphasia or dysarthria (14, 20–24), which would miss some typical signs of cortical, subcortical, or brain stem dysfunction, the possible discriminators of LVOS. Besides, different from the previous 3-ISS, RACE and LAMS scales, which gave a higher weight to motor symptoms, CG-FAST only preserved the simple arm weakness score based upon the consideration that motor symptoms can also occur in lacunar stroke and thus are not good indicators for LVOS, though they strongly relate to the severity of stroke. And neglect was not contained in CG-FAST due to its low inter-rater reliability of evaluation in emergency conditions, though it was reported to be associated with large cortical infarct (14). Instead, we recruited gaze deviation as it could be easily observed by paramedics and was regarded as a typical sign of large artery occlusion in the anterior or posterior circulation (28–30). Last but not least, the method of dichotomy for CG-FAST was simple as no extra ranking information was needed except for the confirmation of presence or absence of symptoms. Moreover, CG-FAST coincided with all tested items as an acronym, which could be easily memorized by emergency paramedics.

Ideally, it is better to use different pre-hospital triage tools with different predictive values for LVOS according to the location of CSC. If a CSC is very close to the patient, then a triage tool with high sensitivity is more appropriate because we need to avoid missing the possible LVOS patients. For distal CSC, a triage tool with high specificity is better so that patients with low possibility of LVOS do not need to waste time on the road. However, this will obviously increase the operational complexity for emergency paramedics, so it is seldom used in real world practice.

Our study has some limitations. First, the scale was derived and validated from a single cohort. The retrospective and observational design inherits potential for bias. Our results need to be confirmed prospectively in larger cohorts. Second, all patients were diagnosed in our center. The predictive value of CG-FAST for LVOS may be different in the pre-hospital settings because it will inevitably mix with stroke mimics or hemorrhagic strokes. Thus, studies performed in a preclinical setting are necessary to generalize our results. Third, the elements derived from the NIHSS were completed at the time of admission to the hospital by experienced neurologists rather than emergency paramedics in the field, although it was proven that NIHSS had good inter-observer reproducibility between physicians and other health personnel (31, 32) and we have already designed our scale to be easily assessed by emergency paramedics. Finally, pre-hospital triage tools could assist emergency paramedics to make rough judgments and transfer appropriate patients to a CSC in a timely manner. But we should realize that no scale could take the place of vessel imaging (33, 34). Therefore, MRA or CTA is better to be performed in all patients suspected of LVOS as soon as possible.

In summary, the CG-FAST scale could be an effective and simple tool to identify patients with high likelihood of LVOS, which might help to select eligible patients for EVT and transfer appropriate patients to a CSC quickly. The utility and accuracy of the CG-FAST scale to predict LVOS in the pre-hospital setting needs to be tested in further prospective studies.

## Ethics Statement

All subjects had given written informed consent prior to the study, and the protocols had been approved by the human ethics committee of the second affiliated hospital of Zhejiang University, School of Medicine. Clinical investigation had been conducted according to the principles expressed in the Declaration of Helsinki.

## Author Contributions

XG and ZC drafted and revised the manuscript, participated in study concept and design, conducted the statistical analyses, analyzed and interpreted the data. ML participated in study concept and design, data interpretation and made a major contribution in revising the manuscript. FS, MZ, CX, and RZ participated in the study design and made contribution in revising the manuscript.

### Conflict of Interest Statement

The authors declare that the research was conducted in the absence of any commercial or financial relationships that could be construed as a potential conflict of interest.
